# Alveolar bone healing in rats: micro-CT, immunohistochemical and molecular analysis

**DOI:** 10.1590/1678-7757-2017-0326

**Published:** 2018-05-29

**Authors:** Jaqueline Suemi HASSUMI, Gabriel MULINARI-SANTOS, André Luis da Silva FABRIS, Ricardo Garcia Mureb JACOB, Alaíde GONÇALVES, Ana Cláudia ROSSI, Alexandre Rodrigues FREIRE, Leonardo Pérez FAVERANI, Roberta OKAMOTO

**Affiliations:** 1 Univ. Estadual Paulista, Faculdade de Odontologia de Araçatuba, Departamento de Cirurgia e Clínica Integrada, Araçatuba, São Paulo, Brasil.; 2 Univ. Estadual Paulista, Faculdade de Odontologia de Araçatuba, Departamento de Ciências Básicas, Araçatuba, São Paulo, Brasil.; 3 Universidade Estadual de Campinas, Faculdade de Odontologia de Piracicaba, Departamento de Morfologia, Piracicaba, São Paulo, Brasil.

**Keywords:** Rats, Gene expression, Osteoprotegerin, Bones and bone, Bone remodeling

## Abstract

Alveolar bone healing after upper incisor extraction in rats is a classical model of preclinical studies. The underlying morphometric, cellular and molecular mechanism, however, remains imprecise in a unique study.

**Objectives:**

The aim of this study was therefore to characterize the alveolar bone healing after upper incisor extraction in rats by micro computed tomographic (Micro-CT), immunohistochemical and real-time polymerase chain reaction (RT-PCR) analysis.

**Material and Methods:**

Thirty animals (*Rattus norvegicus*, Albinus Wistar) were divided into three groups after upper incisors extraction at 7, 14, and 28 days. Micro-CT was evaluated based on the morphometric parameters. Subsequently, the histological analyses and immunostaining of osteoprotegerin (OPG), receptor activator of nuclear kappa B ligand (RANKL) and tartrate resistant acid phosphate (TRAP) was performed. In addition, RT-PCR analyses of OPG, RANKL, the runt-related transcription factor 2 (RUNX2), osteocalcin (OC), osteopontin (OPN), osterix (OST) and receptor activator of nuclear kappa B (RANK) were performed to determine the expression of these proteins in the alveolar bone healing.

**Results:**

Micro-CT: The morphometric parameters of bone volume and trabecular thickness progressively increased over time. Consequently, a gradual decrease in trabecular separation, trabecular space and total bone porosity was observed. Immunohistochemical: There were no differences statistically significant between the positive labeling for OPG, RANKL and TRAP in the different periods. RT-PCR: At 28 days, there was a significant increase in OPG expression, while RANKL expression and the RANKL/OPG ratio both decreased over time.

**Conclusion:**

Micro-CT showed the newly formed bone had favorable morphometric characteristics of quality and quantity. Beyond the RUNX2, OC, OPN, OST, and RANK proteins expressed in the alveolar bone healing, OPG and RANKL activity showed to be essential for activation of basic multicellular units during the alveolar bone healing.

## Introduction

Given the search for a favorable bone and an ideal support for dental implant placement, understanding the alveolar bone healing in preclinical studies is crucial. Bone is a dynamic tissue, where bone cells drive the molecular and cellular mechanisms involved in the bone healing^1^ ^,^ ^12^. These cells act together signaling molecules to maintain the bone turnover^28^. Also, the mechanisms of development and maintenance of bone occurs constantly, since local factors such as mechanical stimulation and systemic factors can interfere in this process^29^. In ideal conditions, bone turnover is balanced by formation and resorption, allowing the maintenance of bone mass and ensuring calcium and phosphate levels^27^. Therefore, the characterization of the dynamic process of bone to replace an extracted tooth is a topic of special interest in Dentistry^4^ ^,^ ^5^.

Alveolar bone healing after tooth extraction has been analyzed in many experimental and clinical conditions. A classical model used to study bone healing after upper incisor extraction in rats is to describe the alveolar healing in three different phases^22^ ^,^ ^23^: first, coagulum formation and cells proliferation from connective tissue; second, connective tissue formation and healing; finally, ossification phase completed with 28 days, and corresponding to 64 days in humans^22^ ^,^ ^23^. Okamoto and de Russo^22^ (1973) defined the dynamic of these events showing an important proteins activity during the 14^th^ day after tooth extraction. Another similar histological study revealed that this process is complete at 28^th^ day with the alveolar socket almost totally filled with bone^23^. In addition, described that the alveolar socket has the crest remodelation and the gingival epithelium regeneration in the end of this process ^23^.

The advent of Molecular Biology raises questions regarding the molecular aspects of bone healing beyond the histological studies, in particular about identifying genes responsible for proteins synthesis involved in the mechanisms of bone healing after tooth extraction. Moreover, the gene expression can be related to the immunohistochemical staining in different areas of the alveolar bone^7^ ^,^ ^8^, which also results in the morphometric parameters evaluated by micro-CT during the alveolar bone healing^30^. In addition, physiologic conditions such as osteoporosis, uncontrolled diabetes and hypertension have been associated with impaired bone metabolism^6^ ^,^ ^20^. All information analyzed in these compromised bone can be compared with the normal condition, in order to provide the morphometric and cellular alterations that influence a favorable bone healing^12^.

Additional to the proteins involved in the bone healing, such as RUNX2, OST, OC, and OPN, the OPG and RANKL are members of the tumor necrosis factor family that are signaled during the cellular responses of bone remodeling^2^. The ratio of OPG to RANKL expression provides an indication whether tissue response tends to bone formation with a predominance of OPG or bone resorption with increase of RANKL^2^ ^,^ ^16^. Thus, a balanced bone remodeling occurs when levels of OPG and RANKL are similar^14^ ^,^ ^24^. Describing how the genes responsible for protein production are expressed during the different steps of the bone healing is important, specifically if, at 14^th^ day after tooth extraction, these genes are overexpressed during the alveolar bone healing as previous suggested^22^ ^,^ ^23^.

Therefore, the aim of this study was to evaluate the morphometric aspects using micro-CT, performing a volumetric assessment of the newly formed bone during the alveolar bone healing in rats, besides the immunostaining of OPG, RANKL, and TRAP, and the messenger RNA (mRNA) expression of OPG, RANKL, RUNX2, OC, OPN, OST and RANK.

## Material and methods

### Study design and ethics

This research was approved by the Ethics Committee for Animal Use from Faculdade de Odontologia de Araçatuba, UNESP – Univ. Estadual Paulista, Brazil (number process 00123-2013).

We used 30 adult (6 months old) male rats (*Rattus norvegicus*, Albinus Wistar) with an average body weight of 275 g ±25 g. The animals were divided into three groups according to their time of upper right incisor extraction:

Group I - Analyzed 7 days after tooth extraction.

Group II - Analyzed 14 days after tooth extraction.

Group III - Analyzed 28 days after tooth extraction.

In each group with 10 animals, being 5 for micro-CT and immunohistochemical analysis and others 5 for RT-PCR analysis.

The sample number was elected by power test analysis in the website http://www.lee.dante.br. Level of significance of 5% and power test of 95% were adopted, and it was suggested four animals *per* group. Thus, with a possible animal loss, it was used five *per* period of analysis.

The animals were kept in cages in an environment at a stable temperature (22±2°C) and a controlled light cycle (12 h light and 12 h dark). The animals were fed a ground solid diet and powder diet 14 days after surgery (Anderson & Clayton SA - Abbot Laboratórios do Brasil Ltda., São Paulo, SP, Brazil) with water *ad libitum*, except during the 12 h prior to surgery.

### Tooth extraction

Surgery was performed under sedation via an intramuscular injection of xylazine hydrochloride (0.03 ml *per* 100 g body weight; Coopers Brasil Ltda, Cotia, SP, Brazil), to promote muscle relaxation, and ketamine hydrochloride (0.07 ml *per* 100 g body weight; Fort Dodge Animal Health, IA, USA), to induce anesthesia. The anterior portion of the right maxilla was disinfected with iodized polyvinylpyrrolidone (PVP Topic 10%, Riodeine – Indústria Farmacêutica Rioquímica Ltda., São José do Rio Preto, SP, Brazil). Animals had their gingival mucosa detached using specific retractors dislocation; subsequently the upper right incisor was extracted using specially adapted forceps. The gingival mucosa was sutured with 4-0 polyglactin 910 suture thread (Johnson & Johnson, São José dos Campos, SP, Brazil). The tooth extraction was performed according to previous studies^16^ ^,^ ^22^ ^,^ ^23^.

### Micro-CT analysis

At 7, 14, or 28 days after tooth extraction, 15 animals (n=5 *per* group) were euthanized by anesthesia overdose (pentobarbital sodium, 100 mg/kg). The right maxilla was removed and fixed in 4% paraformaldehyde solution and 10% 0.1 M phosphate buffer (pH 7.4), after 48 hours in fixation solution.

The middle third of the alveolar sockets were scanned in the direction from apical to cervical in the longitudinal plane using SkyScan Model 1172 (Bruker, Kontich, Belgium) microtomography. The tube current was 165 uA and peak voltage was 60 kV. Image Pixel Size was 9.92 um. The filter to correct beam hardening was Al 0.5 mm, and the frame averaging was 4 and rotation step was 0.6 deg.

After scanning, the images were imported into NRecon Reconstruction software (SkyScan, Leuven, Belgium) for reconstruction in grayscale, presenting x-ray attenuation coefficients with values related to bone structure. Attenuation coefficients were obtained using calibration values for the aqueous medium (formaldehyde solution 10% and 0.1 M phosphate buffer, pH 7.4).

After the bone three-dimensional reconstruction, the morphometric parameters were measured using CT-Analyzer software (SkyScan, Leuven, Belgium): bone volume (BV; mm^3^), percentage of bone volume (BV/TV; %) in relation to the total measured area, trabecular thickness (Tb.Th; mm), trabecular separation (Tb.Sp; mm), and percentage of total bone porosity (Po-tot; %). The morphometric parameters were three-dimensionally calculated.

The segmentation was standardized in the CT-Analyzer software (SkyScan, Leuven, Belgium) after the micro-CT analysis. The values used in segmentation were selected to eliminate artifacts and to keep the bone structure. The greyscale threshold ranged from 70 to 255 for all pieces, in an interval from 0 to 255.

**Figure 1:**
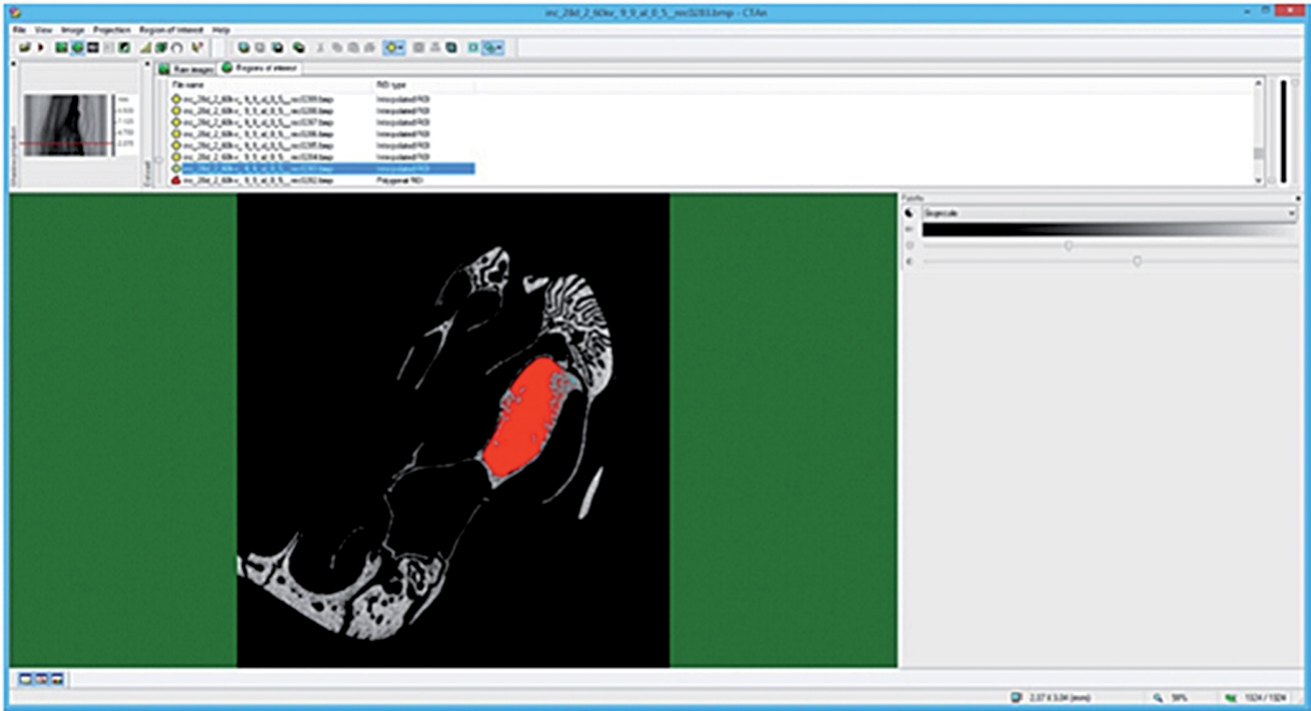
Micro-CT evaluation of alveolar socket

**Figure 2:**
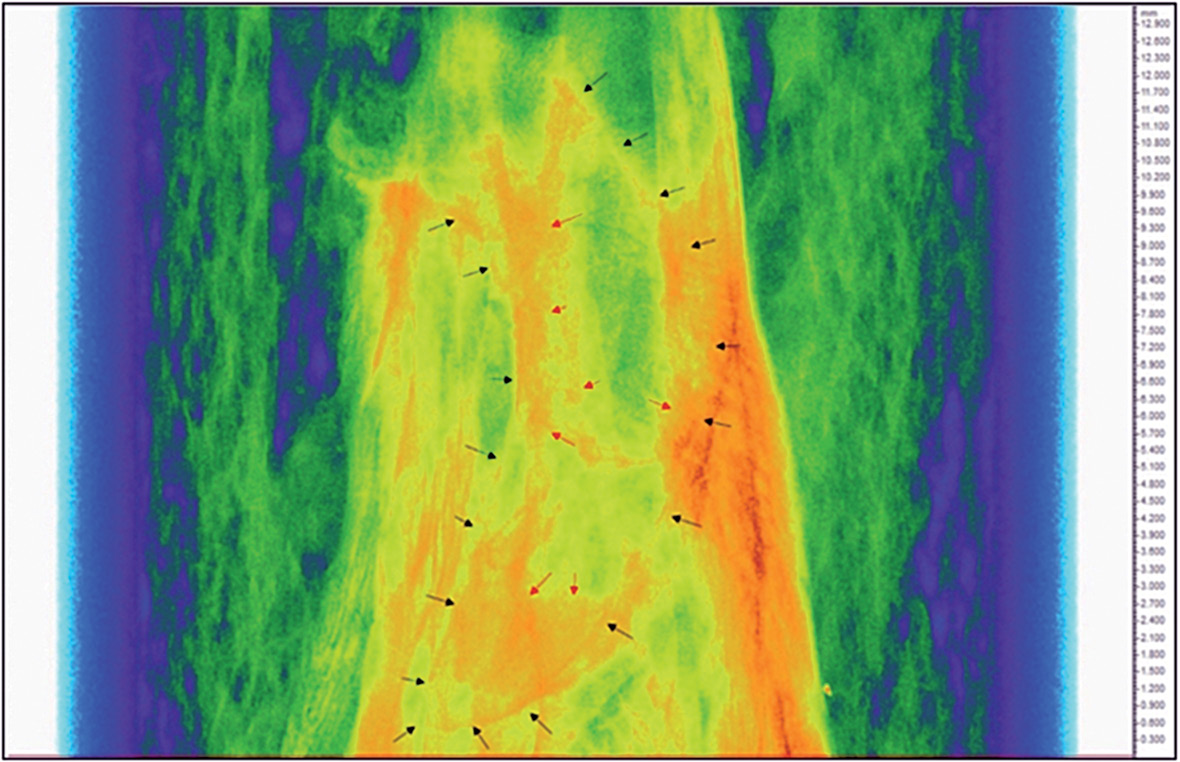
Micro-CT evaluation of alveolar socket

**Figure 3:**
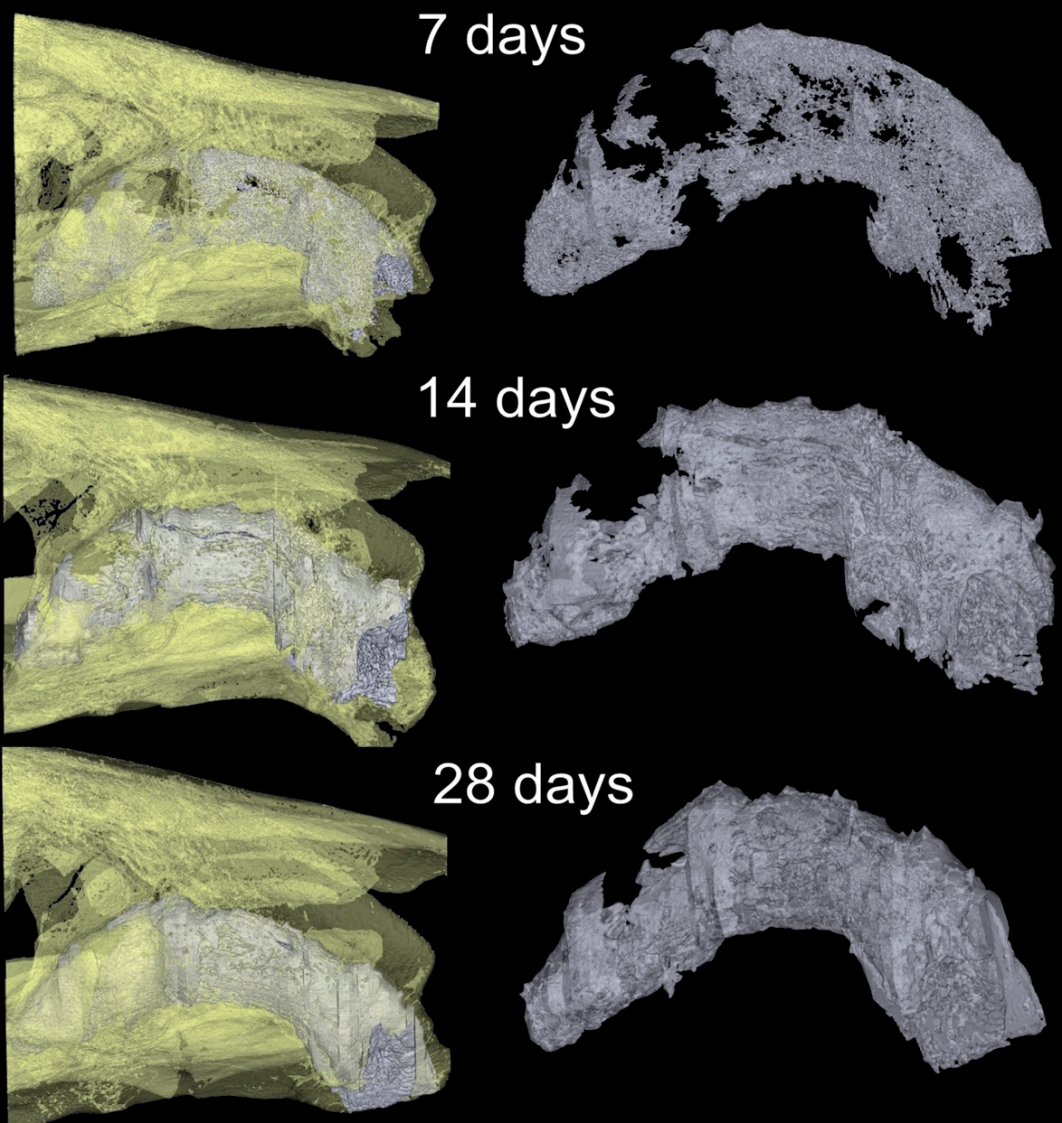
Three-dimensional images of alveolar socket

Due to the irregular alveolar socket morphology, the region of interest (ROI) was standardized using the total number of slices along the middle third of the socket. After determining the ROI, using the interpolated ROI tool, images were converted to grayscale for the three-dimensional calculation, which was performed by the software. (Figures 1 and 2). The Materialise MIMICS Research v18 software (Materialise NV, Leuven, Belgium) was used to create a three-dimensional surface model from Micro-CT of each group (Figures 3).

Micro-CT analysis using CT Analyzer software (SkyScan, Leuven, Belgium). Coronal plane. The area outlined in red indicates the region of interest (ROI), which was positioned within the alveolar socket undergoing bone healing

Region for analysis of middle third of the socket observed using the color filter in the CT Analyzer software (SkyScan, Leuven, Belgium). Sagittal plane. Black arrows delimit the contour of the alveolar socket. Red arrows delimit the area of bone formation

Three-dimensional surface models of the socket observed using the Mimics software (Materialise, Leuven, Belgium). Sagittal plane. White color represents the bone formation in the alveolar socket healing

The Micro-CT analysis followed the guide for evaluation of bone microarchitecture in rats using computed microtomography^3^.

### Sample preparation and histological analysis

Subsequently, the same bones used in the micro-CT analysis were washed for 24 h in running water and decalcified in 10% EDTA for 6 weeks. They were washed for 24 h, dehydrated through an alcohol sequence, cleared in xylene and embedded in paraffin (Merck, Kenilworth, NJ, USA). Sections (5-um thick) were cut with a microtome and mounted on glass slides. Microtome cuts were intended for histological and immunohistochemical analysis. The hematoxylin and eosin stained slides were captured using a Nikon microscope (Eclipse 80i, Shinagawa, Tokyo, Japan). The morphology of the bone tissues obtained were qualitatively evaluated, establishing a comparison between the groups. The slides were photomicrographs magnified from the originals by 6.3x.

### Immunohistochemical analysis

Previously to the immunohistochemistry, endogenous peroxidase activity was inhibited by the sections incubating in hydrogen peroxide. Sections were subjected to antigen retrieval with citrate phosphate buffer (pH 6.0). In order to evaluate cellular responses during bone remodeling, primary antibodies were used against OPG, RANKL and TRAP, polyclonal antibodies, produced in goat (Santa Cruz Biotechnology, Dallas, TX, USA).

The signal was detected using the immunoperoxidase method with a biotinylated anti-goat secondary antibody raised in rabbit (Pierce Biotechnology, Life Technologies Corporation, Grand Island, NY, USA), an avidin and biotin amplifier (Vector Laboratories, Burlingame, CA, USA) and diaminobenzidine (Dako, Carpinteria, CA, USA) as a chromogen. After the diaminobenzidine color reaction, sections were counter-stained with Harris hematoxylin, a counterstaining that allows having the cytoarchitecture reference of the alveolar socket evaluated. Expression of OPG, RANKL, and TRAP proteins was semi-quantitatively evaluated, through a visual evaluation. The evaluations were made under the same conditions and by the same evaluator. The examination was performed in the middle third of the alveolar socket, in a semi-quantitative way, by assigning different “scores” according to the area of positive immunostained cells. Therefore, score 0 represents absence of immunostaining; score 1 denotes mild immunostaining and represents less than 25% of the area; score 2 represents moderate immunostaining and up to 50% of the area; score 3 is intense immunostaining and more than 75% of the area, according to previous studies^8^ ^,^ ^18^ ^,^ ^19^ ^,^ ^25^. The representation of the immunolabeling data was performed through the most frequency score attributed to the animals of each period of evaluation.

### Molecular analysis

Fifteen animals, 5 *per* group at 7, 14, and 28 days after extraction, were submitted to sedation with xylazine hydrochloride (0.03 ml *per* 100 g body weight; Coopers Brasil Ltda., Cotia, SP, Brazil), to promote muscle relaxation, and ketamine hydrochloride (0.07 ml *per* 100 g body weight; Fort Dodge Animal Health, IA, USA), to induce anesthesia. The right maxilla was collected; thereafter the alveolar bone of socket was separated. Each fragment was washed in phosphate buffer solution and frozen in liquid nitrogen. Total RNA was extracted using Trizol reagent (Life Technologies Invitrogen, Carlsbad, CA, USA).

Reverse transcription polymerase chain reaction (RT-PCR) was performed to assess the expression of osteoprotegerin (OPG), receptor activator of nuclear factor kappa B ligant (RANKL), runt-related transcription factor 2 (Runx2), osteocalcin (OC), osteopontin (OPN), osterix (OST), and receptor activator of nuclear factor kappa B (RANK). The rat genes and the TaqMan Gene Expression Assays (Applied Biosystems, Foster City, CA, USA) of the primer/probe sets used were: OPG (Tnfrsf11b, Rn00563499_m1), RANKL (Tnfrsf11, Rn00589289_m1), RUNX2 (Runx2, Rn01512298_m1), OC (Bglap, Rn0056386_g1), OPN (Spp1, Rn00681031_m1), OST (Sp7, Rn02769744_s1) and RANK (Tnfrsf11a, Rn00589289_m1).

After determining the integrity, purity, and concentration of the RNA, cDNA was made using 1 μg of RNA in a reverse transcriptase reaction (M-MLV reverse transcriptase; Promega Corporation, Madison, WI, USA). RT-PCR was performed using a detection system for RT-PCR CFX96 (Bio-Rad Laboratories, Philadelphia, PA, USA) with the SybrGreen system (Applied Biosystems, Warrington, UK) under the conditions: 50°C (2 min), 95°C (10 min) and 40 cycles of 95°C (15 s), 60°C (1 min), followed by a standard denaturation curve. Relative gene expression was calculated in reference to the expression of proteins ribosomal mitochondrial and normalized the gene expression of the alveolar bone at the different experimental periods (ΔΔCT method). Assays were performed in quadruplicate.

### Statistical analysis

Micro-CT data were subjected to the Shapiro-Wilk normality test, which showed homogeneity for some parameters: BV, BV/TV, Tb.Th, and Po-tot, whichever is the parametric test ANOVA-1 factor and those who showed statistical significance, the Holm-Sidak post-test was applied. The Tb.Sp parameter showed heterogeneity, as indicated by the nonparametric Kruskal-Wallis test and a post-test using the Dunn method. RT-PCR data were compared using the nonparametric Kruskal-Wallis test and the Shapiro-Wilk post-test, considering statistical significance p<0.05. Only RANK were subjected to parametric test ANOVA-1 factor and the Shapiro-Wilk post-test, considering statistical significance p<0.05. Immunolabeling data were compared using the nonparametric Kruskal-Wallis test and the Shapiro-Wilk post-test, considering statistical significance p<0.05. All tests considered a significance level of 5%. The statistical program used was SigmaPlot 13.0 (Scientific Data Analysis and Graphing Software, San Jose, CA, USA).

## Results

### Micro-CT analysis

**Figure 4:**
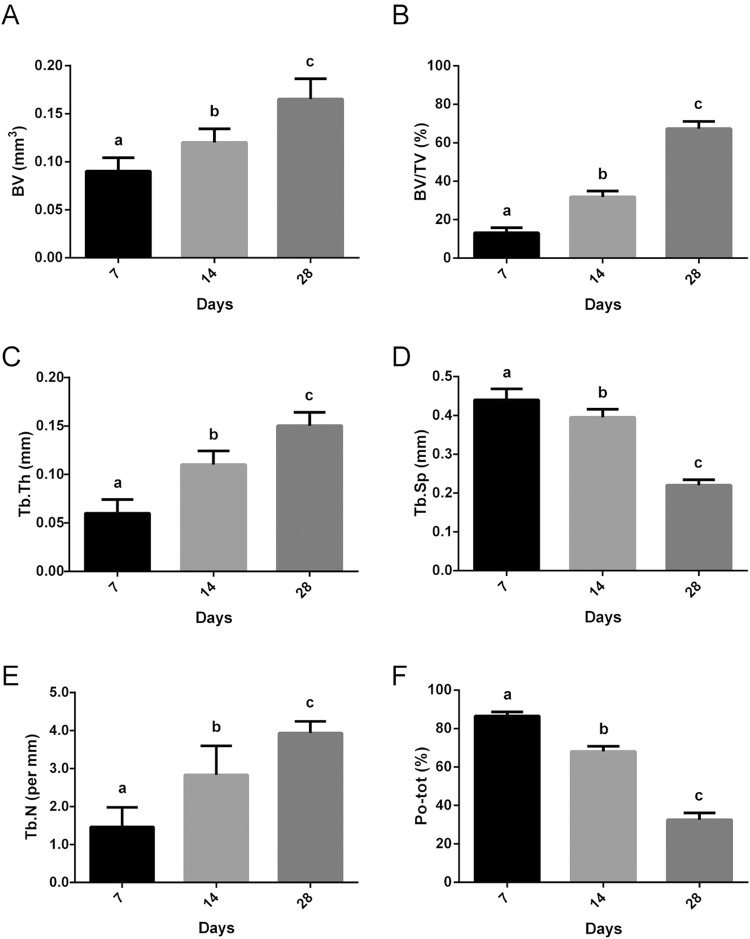
Morphometric results

BV and BV/TV showed a gradual increase throughout the time analyzed (Figures 4A and 4B), with an average of 0.08 mm, 0.12 mm and 0.17 mm in BV and 11.29%, 29.58%, and 64.57% in BV/TV at 7, 14, and 28 days, respectively. Comparison between the periods showed a significant increase for BV (p=0.015, Holm-Sidak) and BV/TV (p<0.001, Holm-Sidak). Tb.Th also progressively increased over time (Figure 4C), with the highest values at 28 days (0.163 mm ±0.01) and the lowest values at 7 days (0.07 mm ±0.005) (p<0.001, Kruskal-Wallis). Consequently, the opposite was observed in Tb.Sp and Po(tot). For Tb.Sp, the highest values were seen at 7 days, and then they gradually decreased over time, with the lowest values at 28 days (Figure 4D). For Tb.Sp, median values were 0.46 mm at 7 days, 0.41 mm at 14 days, and 0.21 mm after 28 days (p<0.05, Kruskal-Wallis). For Tb.N the highest values were at 28 days (3.9 *per* mm), followed by 14 days (2.86 *per* mm) and the lowest at 7 days (1.54 *per* mm) (p <0.05, Kruskal-Wallis, Figure 4E). For Po-tot, values were 88.69% ±3.1 at 7 days, 70.40% ±9.45 at 14 days, and 35.32% ±7.88 at 28 days (p<0.001, Holm-Sidak, Figure 4F). Since 7 days until the 28 days, the three-dimensional images revealed the bone volume and trabecular thickness progressive increasing over time in the alveolar socket healing.

Mean values and standard deviation of BV, BV/TV, Tb.Th, Tb.Sp and Po-tot. BV, BV/TB and Tb.Th increased progressively over time. Consequently, a gradual decrease of Tb.Sp and Po-tot was observed. The different letters (a, b and c) indicate significant statistical difference (p<0.05) between the groups

### Histological analysis

**Figure 5:**
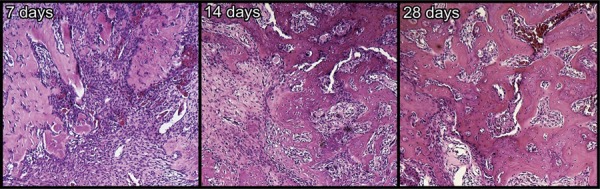
Histology

The histology of the middle third of the alveolar socket at 7, 14, and 28 days after extraction are shown in Figure 5.

Histological images of the medium third of the alveolar socket at 7, 14, and 28 days after extraction. Hematoxylin and eosin staining (63x magnification)

At 7 days, the presence of blood clot into the alveolar socket was observed. A cellular proliferation was noted (fibroblasts, endothelial cells and macrophages), originated from remnants of periodontal ligament. In middle and apical thirds, in some areas, it was possible to detect no organizing tissue, with some immature trabecular bone. The middle third of this period of evaluation shows the presence of initial bone formation with great quantity of inflammatory cells and fibroblasts, with the predominance of great part of the alveolar socket.

At 14 days, the bone trabecular in maturation filling great part of the alveolar socket was detected. Bone formation was observed specially close to middle and cervical thirds. Rare regions with blood clot were observed in this period. The middle third of the alveolar socket representing the group of 14 days shows great quantity of trabecular bone in this portion of the alveolar socket.

At 28 days, mature bone trabecular filling great part of the alveolar socket was observed. It is important to highlight the presence of osteocytes into the trabecular bone, which presented thicker and had well-defined morphology. Small islands of connective tissue were perceived among the newly trabecular bone.

There was no signal of inflammatory response that could be associated to alveolitis process.

### Immunohistochemical analysis

**Figure 6:**
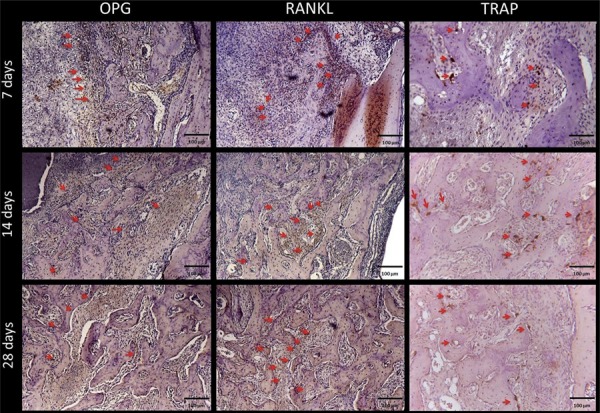
Immunohistochemical staining

Immunostaining of OPG and RANKL protein at 7, 14, and 28 days after extraction are shown in Figure 6. Positive signal of OPG protein was visualized in connective tissue cells, osteoblasts, and osteocytes around and inside the newly trabecular bone. For RANKL, positive signal was in the connective tissue cells and osteoblasts around the newly formed trabecular bone, and in osteocytes trapped in the newly bone. Qualitatively, at 7 days, OPG expression was moderate (2) in connective tissue fibroblasts, while RANKL expression was moderate (2) in osteoblasts surrounding the trabecular bone in the middle and apical thirds of the alveolar socket. At 14 days after extraction, moderate (2) immunostaining for RANKL was noted in osteoblasts, osteocytes, and fibroblasts around the trabecular bone. OPG was moderate (2) in fibroblasts and osteoblasts around the edge of the secreted bone matrix. After 28 days, OPG was moderate (2), while RANKL was moderate to intense (2-3) in osteocytes in the newly trabecular bone. TRAP was detected all periods. TRAP showed mild (1) at 7 days, moderate (2) at 14 days and mild (1) at 28 days.

Representative immunostaining of OPG, RANKL, and TRAP at 7, 14, and 28 days after right upper incisor extraction. OPG and RANKL showed moderate staining all periods analyzed. TRAP was moderate at 14 days and mild at 7 and 28 days. Red arrows indicate intensity of protein tags (63x magnification)

**Figure 7:**
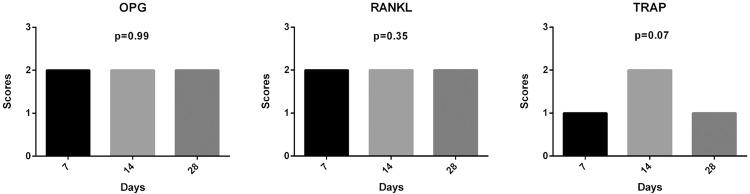
Immunohistochemical results

The scores were submitted to statistical analysis, and there were no differences between the periods for each protein (p>0.05 for comparisons of OPG, RANKL and TRAP) (Figure 7). This result suggests an equilibrium of bone remodeling, represented by OPG (Bone formation) and RANKL (Bone resorption) in all periods of this study.

Graph with the scores and p-value of OPG, RANK and TRAP

### Molecular analysis

**Figure 8:**
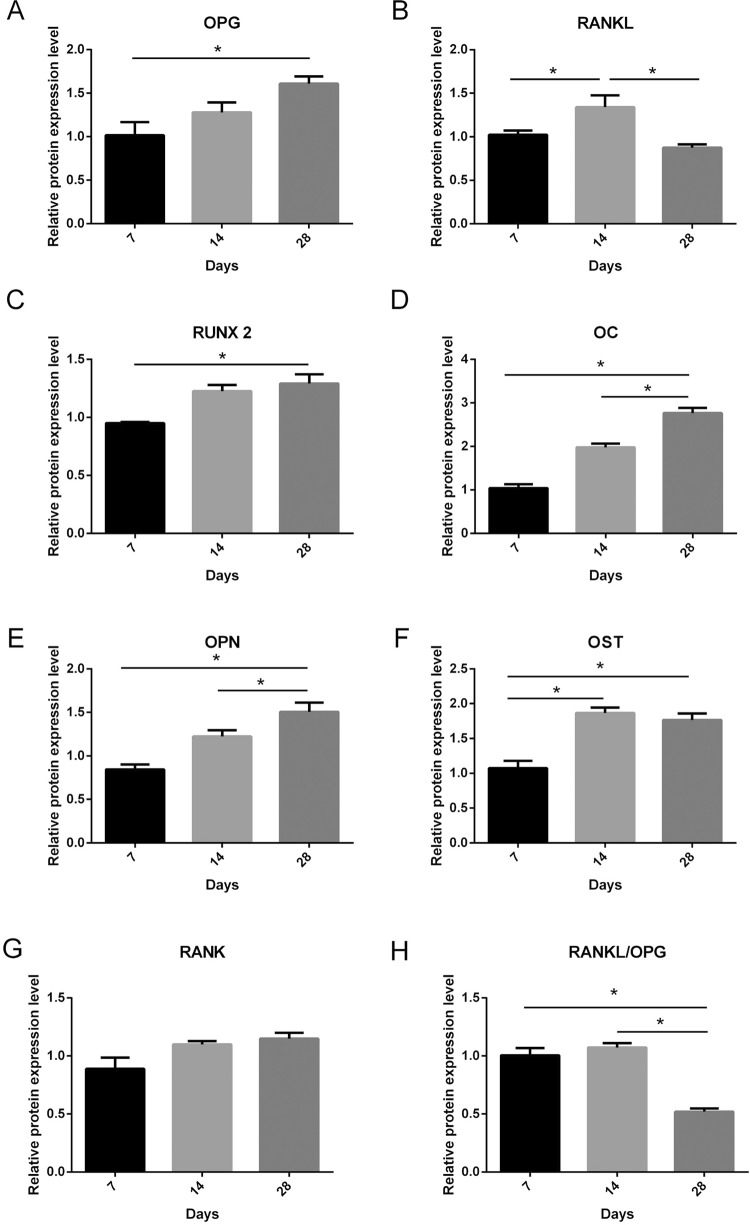
Molecular results

The relative gene expression of OPG showed an increase at 28 days compared with 7 days (p<0.05, Shapiro-Wilk), (Figure 8A). RANKL increased at 14 days compared with 7 and 28 days (p<0.001, Shapiro-Wilk) (Figure 8B). Runx2 expression increased at 28 days compared with 7 days (p<0.05, Shapiro-Wilk) (Figure 8C). OC expression increased at 28 days compared with 7 and 14 days (p<0.05, Shapiro-Wilk) (Figure 8D). OPN expression increased at 28 days compared with 7 and 14 days (p<0.05, Shapiro-Wilk) (Figure 8E). OST expression increased at 28 and 14 days compared with 7 days (p<0.05, Shapiro-Wilk) (Figure 8F). RANK was not significantly increased (p >0.05, ANOVA-1 factor) (Figure 8G). Bone turnover, calculated using the RANKL/OPG ratio, was decreased at 28 days (p<0.05, Tukey test) when compared with 7 and 14 days (Figure 8H).

Graph showing the relative gene expression and standard deviation of OPG, RANKL, Runx2, OC, OPN, OST, RANK, RANKL/OPG. The * indicates significant statistical difference (p<0.05) between time periods

## Discussion

This study characterized the alveolar bone healing after the upper right incisor extraction in rats. The alveolar bone healing showed to be complete at 28 days after surgery, supporting previous studies^15^ ^,^ ^16^ ^,^ ^22^ ^,^ ^23^. This finding was confirmed by micro-CT analysis, which showed the alveolar socket filled with a thick trabecular bone, smaller trabecular separation and reduced bone porous at 28 days after extraction. Also the morphologic data corroborated the qualitative immunohistochemical and molecular results. In the qualitative immunohistochemical, discrete changes in the behavior of OPG, RANKL and TRAP expression during the periods was observed, however, in the quantitative data, after the nonparametric test, there were no statistical differences. Therefore, there were no significant differences in the pattern of the expression of OPG, RANKL and TRAP proteins. It demonstrates that during all of the periods that were evaluated in this study, there was an equilibrium between OPG and RANKL and, consequently, in the remodeling process during the repairing process. In consequence, osteoclast activity represented by TRAP immunolabeling presented similar in all of the periods evaluated in this study. Otherwise, discrete changes observed in the qualitative analysis requires to be considered, specially in association with the molecular and morphometric data, since all methodology need to be regarded together. Thus, this study should be considered when thinking about alveolar bone healing in rats, since this detailed data can support further experimentations. Our morphometric results revealed that, under normal conditions, the newly bone formed displays quite favorable qualitative and quantitative characteristics in this model, in line with the guide for quantitative assessment of bone analysis and supporting that the three-dimensional analyses allow a better characterization of bone^3^.

Alveolar bone healing in rats can be divided into three steps^22^ ^,^ ^23^. First, the formation of a stable fibrin clot allowing cell proliferation that raises the granulation tissue. Second, the granulation tissue forms new connective tissue, which contains mature collagen. Third, this new tissue drives the intramembranous ossification, allowing the alveolar socket to fill itself with the new bone. In this new bone, the basic multicellular unit (BMU) can be well-defined. The BMU is a balance of bone resorption and formation, and osteoclasts and osteoblasts^10^ ^,^ ^17^. Thus, during the alveolar healing, the bone should develop aspects of a mature bone, increasing bone volume and trabecular thickness, causing a decrease of porosity and trabecular space, and it was confirmed in this study. Therefore, these morphological data are an indication of the performance of BMUs towards the alveolar bone healing.

In support of our findings, another study of alveolar socket healing in mice also showed bone volume and trabecular thickness progressive increasing over time by Micro-CT analysis^30^. In addition, the same study confirmed the correspondence between these morphometric aspects and the gene expression profile and histological events^30^. Interestingly, a clinical investigation used the Micro-CT to evaluate the alveolar bone healing with induced laser phototherapy after third molar extraction^26^. In this study was possible to establish a correlation between the morphology by Micro-CT and the histometrical parameters, supporting the acceleration of alveolar bone healing by the laser phototherapy^26^. Another study evaluated the alveolar socket healing in a canine model and demonstrated that periodontal and endodontic pathology can delay the alveolar bone healing after tooth extraction^11^. Thus, it is essential to determinate the alveolar healing nature of a model in order to compare with no physiological conditions. Therefore, the alveolar healing characterization provides knowledge to further researches for a favorable bone healing.

The OPG, RANK and RANKL are of particular interest to bone healing. The dynamic actions of these proteins, especially OPG and RANKL, must be balanced to have bone homeostasis^2^ ^,^ ^16^ ^,^ ^19^. It will result in equilibrium of osteoclasts and osteoblasts in the BMUs^17^. There is strong evidence that suggest a role of OPG/RANK/RANKL system in BMU activity^9^ ^,^ ^28^. Our findings revealed different expressions of OPG and RANKL during the alveolar bone healing. OPG expression increased, and it was significantly elevated at 28 days. RANKL expression was higher at 14 days. These findings are consistent with effective alveolar healing, since should have an initial organization of granulation tissue, following a gradual replacement by connective tissue and bone. This process is proportional to an increase of extracellular OPG, which blocks the bone resorption^13^. Also RANKL that regulates osteoclast activity and bone resorption^9^ had its expression increased after 14 days, succeeding the bone formation. After 28 days, RANKL expression was lower due to inhibition by OPG, which took control of bone formation. TRAP activated by RANKL, represents the osteoclast activity resorption^25^. Consistent with our immunohistochemical data, TRAP showed a higher expression at 14 days. This suggests that a great BMU activity is observed in the middle of the alveolar bone healing, at 14 days after extraction.

Moreover, in relation to the expression of the mRNA, there were differences in the expression of the proteins RUNX2, OST, OC, and OPN along the bone healing^15^ ^,^ ^21^. As expected, the RUNX2 expression increased at 28 days after extraction, performing the differentiation of the pre-osteoblasts into osteoblasts, responsible for the bone formation^21^. For this reason, an increase of the mineral deposition was observed, characterized by the protein OST at 14 and 28 days. In the same way, the bone maturation and organization occurred with the elevation of OC and OPN, which increased with 28 days. This proteins expression detailed the bone healing organization from the granulation tissue to connective tissue until the bone formation.

The question arises about the correlation of mRNA and protein expression in the developing bone. A direct relationship of mRNA and protein is expected. However, in the current study, it was true only for RANKL. The OPG mRNA expression was increased after 28 days, but it did not correspond to the protein expression over the same period. It could be explained by the fact that OPG is a soluble receptor^14^. Consequently, after synthesized by osteoblasts, OPG is released into the extracellular medium, making its detection more difficult by immunohistochemical staining. Despite this, our molecular findings indicate an increase of OPG expression. These data demonstrate the significance of multiple techniques to describe more completely the behavior of a specific protein during the bone healing.

Taken together, these findings demonstrate a significant function of the OPG/RANK/RANKL system in the osteoclasts and osteoblasts response, and BMU activation. Once activated, BMUs provide valuable information of amount and quality to form the bone^17^. It will allow the alveolar socket to fill a thicker and slightly separated trabecular bone. These findings complete previous studies that described the alveolar bone healing in rat ended at 28 days^22^ ^,^ ^23^. Another aspect evaluated was the metabolic activity inside the alveolar socket. At 14 days after extraction, immunostaining revealed high activity of proteins involved in synthesis of the mineralized matrix. Additionally, OPG and RANKL protein showed strong activity at 14 days after extraction, which can be related to higher BMU activity during this time.

In summary, a favorable alveolar bone healing is expected at 28 days after tooth extraction in rats, with equilibrium of RANKL and OPG expression or even the predominance of OPG in the end. The gene expression can be related to the morphometric parameters obtained by Micro-CT analysis. Further studies will be needed to better characterize extracellular matrix formation of the alveolar bone. Moreover, identifying other proteins that are involved in the bone formation and resorption is required. Lastly, we would like to highlight the significance of performing a Micro-CT analysis of bone healing, parallel to protein expression profile and molecular responses. We consider that Micro-CT analysis must be performed to improve the parameters that determine the quality and quantity of the newly formed bone after tooth extraction.

## Conclusions

1) Micro-CT revealed that the newly formed alveolar bone after tooth extraction displays very favorable morphometric characteristics of quality and quantity on this experimental model.

2) Beyond other proteins expressed, the mutual activity of OPG and RANKL is essential for BMUs activation during the alveolar bone healing.
